# When Fever Turns Hemorrhagic in the Maldives: A Case Report of Dengue in a G6PD‐Deficient Young Adult With Hemolysis and Rhabdomyolysis

**DOI:** 10.1155/crdi/2341390

**Published:** 2026-04-03

**Authors:** Rajib Dey, Gunjan Khadka, Thuhufa Ali, Su Su Htun, Ahmed Miqdhaadh, Nestor Arce, Wasin Matsee, Prakaykaew Charunwatthana, Hisham Imad

**Affiliations:** ^1^ Department of Medicine, Indira Gandhi Memorial Hospital, Malé, Maldives, igmh.gov.mv; ^2^ Department of Clinical Tropical Medicine, Faculty of Tropical Medicine, Mahidol University, Bangkok, Thailand, mahidol.ac.th; ^3^ College of Medicine, Jose Maria College Foundation, Inc., Davao, 8000, Philippines, medcol.mw; ^4^ Mahidol Oxford Tropical Medicine Research Unit, Faculty of Tropical Medicine, Mahidol University, Bangkok, 10400, Thailand, mahidol.ac.th

**Keywords:** bleeding diathesis, case report, G6PD deficiency, hemorrhagic fever, Maldives

## Abstract

**Background:**

Dengue is endemic in the Maldives and remains a major public health concern in this small island nation of approximately half a million people. Genetic red blood cell disorders, particularly thalassemia, are common; however, the prevalence of other inherited disorders, such as glucose‐6‐phosphate dehydrogenase (G6PD) deficiency, remains poorly characterized. Despite this, sporadic cases of hemolytic anemia in G6PD deficient individuals have been reported. The presence of G6PD deficiency may alter the clinical course of acute dengue infection, as illustrated in the case presented, and require careful management to prevent complications.

**Case Presentation:**

A 22‐year‐old male presented with fever, headache, nausea, vomiting, and jaundice, which rapidly progressed to include epistaxis and dark‐colored urine. Laboratory findings revealed leukopenia, thrombocytopenia, elevated unconjugated bilirubin, markedly reduced G6PD activity, and elevated creatine kinase levels. While the dengue NS1 antigen test was positive during acute illness, follow‐up serology at 8 months remained negative.

**Conclusions:**

This case depicts the potential for concurrent hemolysis and rhabdomyolysis in G6PD‐deficient patients with dengue in endemic settings. Clinicians should maintain a high index of suspicion for these complications in patients presenting with fever and jaundice, as early recognition through vigilant clinical observation may influence management and clinical outcomes.

## 1. Introduction

Acute undifferentiated febrile illness is a common clinical presentation in tropical regions, with dengue being one of the most common etiologies in high‐burden endemic countries [1]. Similarly, in Maldives, dengue is endemic, characterized by sustained year‐round transmission with periodic large outbreaks, and it remains a leading cause of febrile illness requiring hospitalization [2].

While most individuals with dengue virus infection experience a self‐limiting acute febrile illness and recover fully within a week after symptom onset, a subset of patients may progress to severe disease characterized by endothelial dysfunction and worsening coagulopathy, which can lead to shock and hemorrhage [3]. Severe dengue is more commonly observed in individuals with prior dengue exposure or comorbid conditions, such as diabetes [4]. In addition, inherited red blood cell disorders, such as Glucose‐6‐phosphate dehydrogenase (G6PD) deficiency have been associated with severe dengue [5]. This enzymopathy predisposes individuals to acute intravascular hemolysis and even rhabdomyolysis when exposed to oxidative stressors, such as acute viral infections or exposure to dyes [6, 7].

Here, we report the unmasking of G6PD deficiency, manifesting with hemolysis and rhabdomyolysis, in an adult hospitalized with dengue from an endemic region.

## 2. Case Presentation

A 22‐year‐old male from the Maldives presented to Indhira Gandhi Memorial Hospital in Malé in June 2024 on the second day of illness with sudden high‐grade fever, headache, nausea, and vomiting. Physical examination indicated stable vital signs with no organ‐specific abnormalities. The patient was advised to stay hydrated and was prescribed antipyretics and antiemetics, with instructions to return for follow‐up if symptoms worsened. The following day, his condition worsened with increased vomiting, diarrhea, and abdominal pain. On examination, he appeared febrile, icteric, and dehydrated, with no pallor or cyanosis, and had an ill‐looking appearance with relative bradycardia and normal blood pressure. Additional findings included mild tenderness over the right upper quadrant but no hepatomegaly or rash. The respiratory system was normal, and the cardiovascular system was unremarkable. There was no neurological deficit. Furthermore, there was no history of travel, animal contact, exposure to forested areas, or flash flooding.

Blood tests revealed leukopenia, neutrophilia, and thrombocytopenia. Moreover, there was an elevated unconjugated hyperbilirubin level, mildly raised aspartate aminotransferase, and a mild degree of hyponatremia. The dengue NS1 antigen test (Bioline Dengue Dou, Abbott Diagnostics Korea Inc.) was positive, as shown in Table 1.

**TABLE 1:** Clinical findings and serial laboratory parameters during the course of infection.

Duration after illness onset	Day 3	Day 4	Day 5	Day 6	Day 7	Day 8	Day 9	2 weeks	8 months
Vital signs									
Temperature, °C	39.6	37.8	37.9	38.5	38.9	Afebrile	Afebrile	Afebrile	Afebrile
Pulse, beats per minute	103	86	74		93	85	95		
SBP, mmHg		105			111	107	101		
DBP, mmHg		67			68	65	63		
Warning signs									
Persistent vomiting	+	+							
Abdominal pain		+							
Mucosal bleed		+	+	+	+				
Hemorrhagic manifestations									
Epistaxis		+	+	+	+				
Hemolysis		+							
Upper GI bleeding					+				
Ecchymosis					+				
Hematological profile									
Hemoglobin, g/dL (13.0–17.0)	13.9	13.8	13.5	13.6	12.2	11.1	10.4	9.7	14.2
Hematocrit, % (38.0–50.0)	43.1	34.1	40.1	42.0	37.5	34	32	32.8	46.2
RBC, 10^6^/μL (4.2–5.9)			4.23	4.41	3.9	3.52	3.29		5.15
Reticulocytes, %			0.8						
Leukocytes, /μL (4000–1100)	3800		2880	3760	4490	5130	8250	7670	7620
Neutrophils, % (40–75)	81.2		70.8	57.8	50.5	56.5	55.7	69.1	50.9
Lymphocytes, % (20–45)	9.5		28.4	31.1	33.4	19.6	14.4	17.4	38.1
Monocytes, % (2–10)	13.3		0.4	9.6	13.9	21.6	28.1	12.3	
Basophils, % (0‐1)	0.3		0.0	0.4	0.7	0.5	0.2	1.2	
Eosinophils, % (1–6)	0.7		0.3	1.1	1.5	1.8	1.6	0.0	
Platelets, 10^3^/μL (150–450)	94,000	88,900	48,600	39,400	33,400	44,800	93,300	979,000	330,000
Biochemical profile									
Creatinine, mg/dL (0.6–1.2)	1.12	0.97	0.82	0.95	0.88		0.63	0.8	
Urea, mg/dL (7–20)	32.1	27.82	12.84	19.26	19.26		17.12	17.12	
Total bilirubin, mg/dL (0.1–1.2)	3.9	2.7	1.9	1.5			2.3	0.8	2.0
Direct bilirubin, mg/dL (0.0–0.3)	0.5	0.6	0.6	0.4			0.6	0.3	0.5
Indirect bilirubin, mg/dL (0.2–0.8)	3.4	2.1	1.3	1.1			1.7	0.5	1.5
Albumin, g/dL (3.5–5.0)	7.0	6.70	6.1	5.0			3.5	4.6	5.1
Protein, g/dL (6.0–8.3)	4.5	4.3	3.5	7.80			6.10	8.10	8.30
Aspartate aminotransferase, U/L (10–40)	134	175	385				365	48	33
Alanine aminotransferase, U/L (10–55)	29	33	100				251	74	43
Alkaline phosphatase, U/L (40–130)	52	57	64				92	110	68
Lactate dehydrogenase, U/L (140–280)			2067						
Haptoglobin, (30 to 200 mg/dL)			38						
G6PD, U/L (> 290)			10						87
Creatine kinase, U/L (38–174)			2146	2930	2730	2379	1308	473	
C‐reactive protein, mg/L (< 5.0)	1.7			0.27				0.32	
Serum sodium, mEq/L (135–145)	127	133	134	132	134		132	133	137
Serum potassium, mEq/L (3.5–5.0)	4.3	4.8	4.5	4.8	4.5		4.0	4.3	4.3
Urinalysis									
Color	Straw		Brown					Straw	Straw
Appearance	Clear		Cloudy					Clear	Clear
Specific gravity, (1.005–1.030)	1.012		1.012					1.011	
Protein	1+		2+					‐ve	‐ve
Myoglobin								‐ve	
Red blood cells/HPF	6.5		1.2					1.5	0.1
Casts	‐ve							‐ve	
Serology									
Dengue NS1 antigen	+ve								
Antidengue IgM^†^	‐ve								
Antidengue IgG^†^	‐ve								
Dengue ELISA IgM^‡^									‐ve
Dengue ELISA IgG^‡^									‐ve

*Note:* RDW: red cell distribution width, NS1: nonstructural protein 1, IgM: immunoglobulin M, IgG: immunoglobulin G.

Abbreviations: DBP, diastolic blood pressure; G6PD, glucose‐6‐phosphate dehydrogenase; MCV, mean corpuscular volume; RBC, red blood cells; SBP, systolic blood pressure.

^†^Bioline Dengue Duo (Abbott Diagnostics Korea Inc).

^‡^Panbio Dengue Capture IgM/IgG ELISA (Abbott Diagnostics Korea Inc).

**FIGURE 1:**
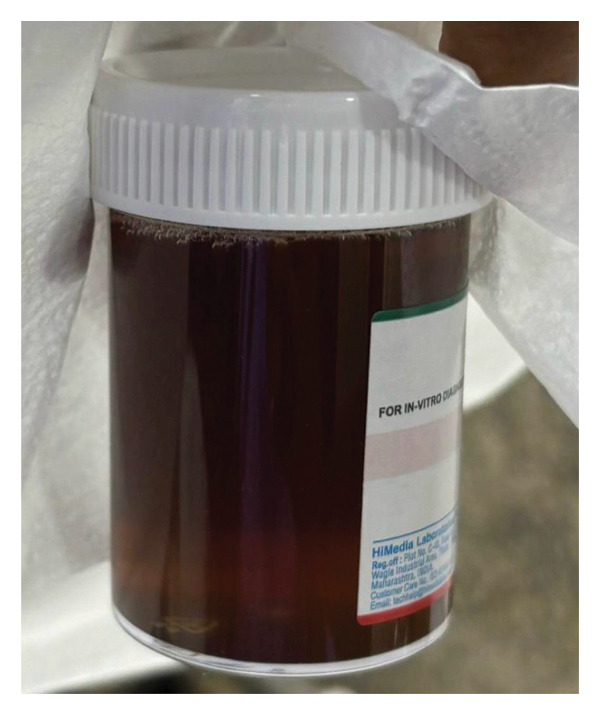
Urine collected on the fourth day of illness, following the onset of symptoms.

The patient was admitted with a provisional diagnosis of dengue with warning signs. Within hours of admission, bleeding diathesis started with epistaxis, and later that day, he reported passing dark‐colored urine, a symptom he had never experienced in the past, as shown in Figure 1.

This presentation required differentiation between myoglobinuria, hemoglobinuria, and hematuria, particularly given the concurrent elevation of creatinine kinase (CK) levels and the sudden change in urine color. However, the presence of hemorrhagic manifestations and underlying G6PD added complexity to explain the sudden change in urine color during a febrile illness.

With the elevation of CK more than five times the upper limit of normal range, the presented case was managed with as rhabdomyolysis, given the concurrent presence of acute kidney injury and dark‐colored urine. Management included intravenous fluid therapy to support renal perfusion and prevent further kidney injury. The urine color gradually normalized as the patient’s CK levels returned to within normal limits, and no further bleeding episodes were observed.

## 3. Discussion

This case highlights a complex clinical presentation of an adult male with underlying G6PD deficiency who developed hemorrhagic manifestations and rhabdomyolysis during an acute febrile illness in a dengue‐endemic setting. Following confirmation of the low G6PD enzyme activity, a more detailed history was obtained to explore prior episodes of hemolysis during acute illness, including influenza‐like infections. No such previous events were reported. A comprehensive clinical history excluded exposure to recognized oxidative triggers before symptoms onset. The patient denied any history of using medication known to precipitate hemolysis, inducing primaquine, tafenoquine, sulfonamides, dapsone, and nitrofurantoin, and reported no exposure to henna or fava beans. These findings strongly suggest that the hemolytic crisis was precipitated by infection‐related oxidative stress [8].

Although jaundice is uncommon early in dengue virus infection, it may occur later in the illness, particularly during defervescence, reflecting hepatocellular injury, cholestasis, or contributing to hemolysis [9]. However, in tropical regions, acute febrile illness with jaundice in adults more often reflect infections such as malaria, leptospirosis, rickettsial disease, or enteric fever, and these should be actively considered and excluded [10].

In dengue‐endemic tropical regions, diagnosis during the acute febrile phase relies heavily on detection of dengue nonstructural protein 1 (NS1) antigen, using rapid diagnostic tests (RDTs). The antigen testing serves as an important screening tool for early dengue detection, enabling timely clinical decision‐making where access to molecular diagnostics, such as RT‐PCR, may be limited or where turnaround times are longer. In the presented case, NS1 antigen positivity in the absence of detectable antidengue IgM or IgG is most consistent with very early acute infection, as serological responses are often not yet detectable by the third day of illness, particularly during a primary dengue infection. Assay‐related limitations, including reduced sensitivity or isolated NS1 cross‐reactivity with other flaviviruses, may also account for this observation [11, 12]. Nevertheless, when interpreted in the context of the clinical presentation and relevant epidemiological exposure, the presence of NS1 antigenemia remains highly suggestive of acute dengue infection.

Following the initial diagnostic impression, clinicians typically turn to the hematological and biochemical profiles to assess for patterns classically associated with dengue infection and to identify features that may signal atypical disease mechanisms or alternative contributors to severity. In this case, the hematological profile demonstrated several deviations from the classical dengue pattern [13]. Although leukopenia was present early in the illness, it was not accompanied by the profound neutropenia and relative lymphocytosis typically observed during the recovery phase of dengue infection. Instead, neutrophil predominance persisted throughout much of the clinical course. Moreover, despite the presence of thrombocytopenia and hemorrhagic manifestations, there was no clinical or laboratory evidence of dengue‐associated plasma leakage, including hemoconcentration or features of effusions. Plasma leakage is commonly observed during secondary dengue infections and is closely linked to immune‐mediated endothelial dysfunction. The absence of plasma leakage in the presented case supports a primary dengue infection and highlights that clinically significant complications may arise through alternative pathogenic mechanisms, particularly in individuals with inherited anemias [5].

Rhabdomyolysis is a rare manifestation of dengue infection [14]. Despite no evidence of myalgia and, as described earlier, the absence of plasma leakage, elevated CK levels prompted early recognition and management of rhabdomyolysis with intravenous fluid therapy. Nonetheless, in dengue patients with plasma leakage, fluid administration for rhabdomyolysis should be undertaken judiciously with careful clinical monitoring to avoid fluid overload [15].

Several limitations should be acknowledged. Confirmatory dengue testing beyond the acute phase was not performed, including RT‐PCR, viral isolation, or paired serology during defervescence and convalescence. Repeat dengue serology performed 8 months later was negative, which may reflect true absence of seroconversion or limitations in assay sensitivity. Despite these constraints, the case fulfilled the WHO 2009 clinical criteria for probable dengue based on fever with warning signs and a positive NS1 antigen test, which has a high positive predictive value in hyperendemic settings such as the Maldives during early illness. While alternative etiologies of hemorrhagic febrile illness must be considered in the absence of seroconversion, dengue remains the most plausible diagnosis. Chikungunya virus infection cannot be entirely excluded; however, the absence of prominent polyarthralgia or arthritis argues against it. Other viral hemorrhagic fevers, including severe fever with thrombocytopenia syndrome virus, Nipah virus, and Crimean–Congo hemorrhagic fever virus, remain theoretical considerations as no cases have been reported in the Maldives to date.

## 4. Conclusion

In conclusion, this case reflects the importance of clinical judgment when encountering overlapping syndromes in acute febrile illnesses. Dengue remains the most plausible etiology clinicians must remain vigilant for coexisting complications and alternative diagnoses, particularly in endemic regions. Individuals with G6PD deficiency must avoid known hemolytic triggers, including certain drugs, dyes, and foods. In such cases, preventive measures such as vaccination should be taken.

## Author Contributions

Conceptualization, Rajib Dey and Hisham Imad; methodology, Rajib Dey and Hisham Imad; validation, Rajib Dey, Gunjan Khadka, Thuhufa Ali, Su Su Htun, Ahmed Miqdhaadh, Nestor Arce Jr., Wasin Matsee, and Prakaykaew Charunwatthana; formal analysis, Rajib Dey, Gunjan Khadka, Thuhufa Ali, Su Su Htun, Ahmed Miqdhaadh, Nestor Arce Jr., Wasin Matsee, Prakaykaew Charunwatthana, and Hisham Imad; investigation, Rajib Dey, Gunjan Khadka, Thuhufa Ali, Su Su Htun, and Ahmed Miqdhaadh; resources, Rajib Dey, Gunjan Khadka, Thuhufa Ali, Su Su Htun, and Ahmed Miqdhaadh; data curation, Rajib Dey, Gunjan Khadka, Thuhufa Ali, Su Su Htun, Ahmed Miqdhaadh, Nestor Arce Jr., Wasin Matsee, Prakaykaew Charunwatthana, and Hisham Imad; writing–original draft preparation, Hisham Imad; writing–review and editing, Rajib Dey, Gunjan Khadka, Thuhufa Ali, Su Su Htun, Ahmed Miqdhaadh, Nestor Arce Jr., Wasin Matsee, Prakaykaew Charunwatthana, and Hisham Imad; supervision, Nestor Arce Jr., Wasin Matsee, Prakaykaew Charunwatthana, and Hisham Imad; project administration, Rajib Dey and Hisham Imad.

## Funding

This report received no external funding.

## Disclosure

All authors have read and agreed to the final version of the manuscript.

## Ethics Statement

We retrospectively reviewed the clinical data after obtaining approval from the hospital. All data were deidentified and analyzed anonymously, ensuring patient identification was not known. An exemption from ethical review was granted by the National Healthcare Academy (Ref: 137‐B(NHA)/MISC/2025/011, dated January 30, 2025). Ethical review was waived as this study involves a single case report.

## Consent

Informed consent was obtained from the subject involved in the study.

## Conflicts of Interest

The authors declare no conflicts of interest.

## Data Availability

The data are not publicly accessible due to privacy involving patient medical records.
